# Genome-Wide Identification of *Populus* Malectin/Malectin-Like Domain-Containing Proteins and Expression Analyses Reveal Novel Candidates for Signaling and Regulation of Wood Development

**DOI:** 10.3389/fpls.2020.588846

**Published:** 2020-12-22

**Authors:** Vikash Kumar, Evgeniy N. Donev, Félix R. Barbut, Sunita Kushwah, Chanaka Mannapperuma, János Urbancsok, Ewa J. Mellerowicz

**Affiliations:** ^1^Department of Forest Genetics and Plant Physiology, Umeå Plant Science Centre, Swedish University of Agricultural Sciences, Umeå, Sweden; ^2^Department of Plant Physiology, Umeå Plant Science Centre, Umeå University, Umeå, Sweden

**Keywords:** *Populus*, cell wall integrity, malectin domain, malectin-like domain, CBM57, receptor-like protein kinases, *Cr*RLK1L

## Abstract

Malectin domain (MD) is a ligand-binding protein motif of pro- and eukaryotes. It is particularly abundant in Viridiplantae, where it occurs as either a single (MD, PF11721) or tandemly duplicated domain (PF12819) called malectin-like domain (MLD). In herbaceous plants, MD- or MLD-containing proteins (MD proteins) are known to regulate development, reproduction, and resistance to various stresses. However, their functions in woody plants have not yet been studied. To unravel their potential role in wood development, we carried out genome-wide identification of MD proteins in the model tree species black cottonwood (*Populus trichocarpa*), and analyzed their expression and co-expression networks. *P. trichocarpa* had 146 *MD* genes assigned to 14 different clades, two of which were specific to the genus *Populus*. 87% of these genes were located on chromosomes, the rest being associated with scaffolds. Based on their protein domain organization, and in agreement with the exon-intron structures, the *MD* genes identified here could be classified into five superclades having the following domains: leucine-rich repeat (LRR)-MD-protein kinase (PK), MLD-LRR-PK, MLD-PK (*Cr*RLK1L), MLD-LRR, and MD-Kinesin. Whereas the majority of *MD* genes were highly expressed in leaves, particularly under stress conditions, eighteen showed a peak of expression during secondary wall formation in the xylem and their co-expression networks suggested signaling functions in cell wall integrity, pathogen-associated molecular patterns, calcium, ROS, and hormone pathways. Thus, *P. trichocarpa MD* genes having different domain organizations comprise many genes with putative foliar defense functions, some of which could be specific to *Populus* and related species, as well as genes with potential involvement in signaling pathways in other tissues including developing wood.

## Introduction

Plant cells are surrounded by cell walls made of cellulose, hemicelluloses, pectins and structural proteins, with lignin being present in cell types specialized for mechanical support (sclerenchyma) and water transport (xylem). Cell wall biosynthesis needs to be regulated so that its mechanical properties can be adapted to different circumstances according to the signals perceived. It is becoming generally accepted that there is constant feedback from the wall to the protoplast, mediated by different molecular pathways commonly termed cell wall integrity (CWI) signaling (Hématy et al., 2007; reviewed by Wolf and Höfte, 2014; Hamann, 2015; Voxeur and Höfte, 2016; Wolf, 2017; Rui and Dinneny, 2020). Perception of signals external to the protoplast is usually mediated by plasmalemma-localized proteins with various ectodomains. One large group of ectodomain-containing proteins is the receptor-like kinases (RLKs) that allow the plant cells to perceive external cues and transduce them, using a phosphorylation relay, into signals to initiate cellular responses (Gish and Clark, 2011; Engelsdorf and Hamann, 2014). Plant RLKs belong to the RLK/Pelle kinase family, one of the largest gene families in plants with more than 600 members in *Arabidopsis* (Shiu and Bleecker, 2001, 2003). It comprises both RLKs and receptor-like cytoplasmic kinases (RLCKs), and has been divided into 45 subfamilies, including wall-associated kinases, extensin-like RLKs, lectin RLKs, and leucine-rich repeat RLKs. RLCKs are cytoplasmic kinases without a transmembrane domain (TMD) and they recognize signaling molecules intracellularly. The RLKs usually function as heterodimers: one subunit with a large extracellular domain interacts with a ligand, and the other, which has a smaller extracellular domain, stabilizes this interaction and enhances signal transduction (Xi et al., 2019).

Among the different clades of plant RLKs, the *Catharanthus roseus* receptor-like kinase 1-like proteins (*Cr*RLK1Ls) have received significant attention as mediators of CWI (reviewed by Wolf and Höfte, 2014; Li et al., 2016; Franck et al., 2018). The family is conserved in all Streptophytes analyzed so far, including moss and liverwort, indicating its ancient origin (Galindo-Trigo et al., 2016). *Cr*RLK1Ls are characterized by two malectin ectodomains (MDs) forming a malectin-like domain (MLD), a transmembrane helix and a C-terminal intracellular Ser and Thr kinase domain. The *Arabidopsis* genome contains 17 *Cr*RLK1L genes and the majority of them have been functionally analyzed. THESEUS1 (THE1) was the first member to be identified as a mediator of dwarfism and ectopic lignification induced by defects in cellulose biosynthesis (Hématy et al., 2007; Merz et al., 2017). Other members of *Cr*RLK1L family including CURVY1 (CVY1), FERONIA (FER) and ANXUR1 (ANX1) are required for polar cell growth in different cell types. FER, ANX1/2 and BUDDHA’S PAPER SEAL1 and 2 (BUPS1 and 2) participate in sexual reproduction. FER mediates signaling by reactive oxygen species (ROS) and Ca^2+^ during pollen tube reception at the filiform apparatus (Escobar-Restrepo et al., 2007), whereas ANX1/2 together with BUPS1/2 form a receptor complex for RAPID ALKALINIZATION FACTOR (RALF) 4 or 19 in the growing tip of pollen tube and regulate ROS and Ca^2+^ gradients essential for its growth and CWI (Ge et al., 2017). In addition, *Cr*RLK1L proteins are involved in immune responses. FER positively regulates pathogen-associated molecular pattern (PAMP)-triggered immunity (PTI) by facilitating the formation of a receptor complex composed of BAK1-FLS2-FER or BAK1-EFR-FER (Stegmann et al., 2017), whereas ANX1 functions antagonistically in PTI and inhibits effector-triggered immunity (ETI) (Mang et al., 2017). The downstream responses of *Cr*RLK1Ls are diverse and include Rho-GTPases activating NADPH oxidases involved in the production of apoplastic ROS (Foreman et al., 2003; Duan et al., 2010; Denness et al., 2011; Boisson-Dernier et al., 2013), RLCKs (Boisson-Dernier et al., 2015; Du et al., 2016), inhibition of the proton pump AHA1 (Haruta et al., 2014), Ca^2+^ signaling mediated by MLO proteins (Kessler et al., 2010; Meng et al., 2020), as yet unknown Ca^2+^ channels and a signaling cascade via intracellular kinases that eventually activate or repress gene transcription (Franck et al., 2018).

The MLD, which is characteristic of *Cr*RLK1L proteins, and the MD are also found in other types of plant RLKs (Zhang et al., 2016; Bellande et al., 2017). The MD was first identified in the protein called malectin residing in the endoplasmic reticulum of *Xenopus laevis* and other animals, where it monitors protein glycosylation by binding diglucose motifs with α-1,4-, α-1,3- and α-1,2-linkage in glycosylated proteins (Schallus et al., 2008, 2010). However, the crystal structure of MLD in ANX1, ANX2, and FER indicated an absence of the aromatic residues that interact with diglucosides in animal MDs, and suggested different ligand specificities and/or functions of the MLDs in these proteins (Du et al., 2018; Moussu et al., 2018; Xiao et al., 2019). Several peptides from the RALF family have been demonstrated to bind to ectodomains of *Cr*RLK1L proteins in *Arabidopsis*: RALF34 to THE1 (Gonneau et al., 2018), RALF1/17/23/32/33 to FER (Haruta et al., 2014; Stegmann et al., 2017), and RALF4/19 to the ANX1/2-BUPS1/2 receptor complex (Ge et al., 2017). Recently it has been shown that the binding of RALF23 to FER is stabilized by interaction with LORELEI-like-GPI-ANCHORED PROTEINS (LLGs) and the formation of such a heterocomplex is required for PTI signaling (Xiao et al., 2019). Moreover, the ectodomain of FER has been shown to bind to the leucine-rich repeat (LRR) domain of LRR-extensin 1 (LRX1) (Dünser et al., 2019) and to pectin (Feng et al., 2018).

Malectin domain is classified as CBM57 in the CAZy database^1^. Interestingly, the CBM57 family is greatly expanded in the model tree species *Populus trichocarpa* compared to the herbaceous model plant *Arabidopsis thaliana* (Kumar et al., 2019). Moreover, transcript of the CBM57 family members are highly upregulated in developing wood tissues of *Populus tremula* (Kumar et al., 2019) and *Eucalyptus grandis* (Pinard et al., 2015). These data suggest that MD/MLD-containing proteins (subsequently called MD proteins) have important functions in trees. We hypothesize that MD proteins are involved in the regulation of cell wall formation during secondary growth via pathways analogous to those reported for primary growth (Wolf and Höfte, 2014; Hamann, 2015; Li et al., 2016; Wolf, 2017), and that they participate in signaling cascades related to stress responses and developmental processes in trees. To find candidates for receptors active during secondary growth, we first carried out genome-wide identification of *P. trichocarpa* genes with predicted MD and MLD. Second, we used expression datasets from different organs (Sundell et al., 2015; Immanen et al., 2016) and high-resolution expression data for wood developmental zones in *P. tremula* (Sundell et al., 2017) to identify those MD proteins that are expressed during wood biosynthesis, and to classify them according to expression at specific stages of xylogenesis. Finally, we identified co-expression networks for the MD proteins expressed during secondary wall deposition, which include their putative interactors. Our analyses provide a framework to identify CWI monitoring, stress response, and other signaling pathways operating during wood development.

## Materials and Methods

### Identification of *P. trichocarpa* Proteins With Malectin and Malectin-Like Domains

The MD proteins of black cottonwood (*P. trichocarpa* Torr. and A. Gray) were identified by Basic Local Alignment Search Tool (BLAST) (Altschul et al., 1990) searches in the genome browser of the PopGenIE database^2^ containing *P. trichocarpa* genome assembly v3.0, using as baits the *P. trichocarpa* proteins containing Pfam domains 11721 and 12819, corresponding to MD and MLD, respectively, retrieved from the Pfam database^3^ (El-Gebali et al., 2019). The same approach was applied to *A. thaliana* using the TAIR database (v10.0) for BLAST searches^4^. The BLASTP tool of a high-performance sequence aligner DIAMOND was used in –unal 0, –evalue 1e-05, –max-target-seqs 4000, and –more-sensitive mode, other parameters were kept as default (Buchfink et al., 2015). All other web-based tools were used in default mode as per developers’ recommendations. The presence of MDs/MLDs in the proteins selected for both *P. trichocarpa* and *A. thaliana* was confirmed using the CDvist web tool^5^ (Adebali et al., 2015), which also served to identify other conserved domains in these proteins. The amino acid sequence lengths, molecular weights, isoelectric points and indices of protein stability of the putative proteins were calculated using the ProtParam tool provided on the ExPASy website^6^. The presence of signal peptides and subcellular localization were predicted with the SignalP 4.1 server^7^ (Petersen et al., 2011) and DeepLoc-1.0 server^8^ (Armenteros et al., 2017), respectively. The exon-intron organization of the *PtMD* genes was determined using the PopGenIE GBrowse tool^9^ and their localization was mapped to *P. trichocarpa* chromosomes using the chromosome-diagram tool^10^. Assignment of a gene to a gene cluster on each chromosome was based on the definition of Holub (2001).

### Phylogenetic Analysis and Classification of the MD Proteins of *P. trichocarpa*

All *Pt*MD proteins identified were classified into clades based on phylogenetic analysis with *A. thaliana*. The amino acid sequences were aligned by MUSCLE^11^ and phylogenetic trees were constructed using the neighbor-joining (NJ) method in the MEGA7 software package with a bootstrap test with 1000 replicates (Kumar et al., 2016).

To identify the conserved residues in MD and MLD regions of poplar MD proteins, these regions were aligned with reference sequences using Jalview Version 2 (Waterhouse et al., 2009) with the MAFFT option (Katoh et al., 2005).

To evaluate evolutionary conservation of *MD* genes across tree species, we have extracted protein sequences for *Eucalyptus grandis* v2.0, *Malus domestica* v1.0, *Salix purpurea* v1.0, *Theobroma cacao* v2.1, *Citrus sinensis* v1.1, *Prunus persica* v1.0 and *Betula pendula* v1.0 from Phytozome genome portal^12^ using BLAST (with same parameters as stated in the section above) and *Pt*MDs as query sequences. The protein sequences of resulting hits and the MD proteins of *P. trichocarpa* and *A. thaliana* were used to generate a phylogenetic tree using one click method described in https://ngphylogeny.fr (Lemoine et al., 2019). The phylogenetic tree and other detailed method descriptions can be found at ftp://plantgenie.org/Publications/Kumar2020/Phylogeny.

### Expression Analysis of *PtMDs* in Developing Leaves and Wood

Developing leaves (leaf number 8, 11, 21 and 23) and developing wood including cambium/phloem and xylem depositing secondary walls were collected from 10 weeks old hybrid aspen (*Populus tremula* L. *x tremuloides* Michx.) grown in the greenhouse. The cultivation conditions and RNA extraction protocols were as described in Ratke et al. (2018). Between five and ten biological replicates of each sample were sequenced using Illumina HiSeq-PE150 platforms of Novogene Bioinformatics Technology Co., Ltd. (Beijing). Quality control and mapping to *P. trichocarpa* transcriptome v3.0 of leaf 8 and 11 samples were performed by Novogene. Other samples had RNA-Seq raw data filtered using FastQC (v0.10.1^13^). rRNA reads were removed using SortMeRNA v1.8 (Kopylova et al., 2012). Low-quality reads were removed using Trimmomatic v0.27 (Lohse et al., 2012) with a sliding window of 5 bp, minimum quality score of 20, minimum read length of 50 bp, minimum leading read quality of 20 and a custom clipping file containing all Illumina adapters. The preprocessed reads were mapped to v3.0 of the *P. trichocarpa* transcriptome (retrieved from PopGenIE see footnote 2) using Kallisto (v0.43.1) with default parameters (Bray et al., 2016). The raw counts were normalized separately for each experiment using Variance Stabilizing Transformation (VST) in R (v3.4.0; R Core Team, 2014) using the Bioconductor (v3.4; Gentleman et al., 2004) DESeq2 package (v1.16.1; Love et al., 2014). Then the VST data were merged together using a sample-based median centering approach as described by Kumar et al. (2019; the R scripts are available at https://github.com/UPSCb/UPSCb/tree/master/manuscripts/Kumar2018). Mean VST data for *PtMD*s were displayed using ComplexHeatmap with default parameters and using the tissue with a peak of expression for each gene as a categorical variable for clustering (Gu et al., 2016). The tissue/organ specificity score *tau* - a score ranging from 0 (ubiquitous expression) to 1 (tissue/organ specific expression), as detailed in Yanai et al. (2005) was calculated for each *PtMD* gene. The customized R scripts used to calculate *tau* are available at https://github.com/UPSCb/UPSCb/tree/master/manuscripts/Kumar2018. The raw RNA-Seq data for this study have been deposited in the European Nucleotide Archive (ENA) at EMBL-EBI under accession number PRJEB41170^14^.

### Expression of *PtMDs* in Different Organs of *Populus*

RNA-Seq datasets of expression values in different tissues/organs of outdoor and greenhouse grown aspen (*P. tremula* L.) and hybrid aspen (*P. tremula* L. x *tremuloides* Michx., clone T89) are available from the PlantGenIE website (Sundell et al., 2015). Data for secondary tissues of greenhouse grown T89 hybrid aspen are detailed by Immanen et al. (2016). Raw data for all biological replicates of each sample (min. = 3) were preprocessed as described in the section above except that the reads were aligned to the *Populus trichocarpa* genome using STAR and quantified using HTSeq. Other steps of quality assessment and filtering are explained above and available at: http://www.epigenesys.eu/en/protocols/bio-informatics/1283-guidelines-for-rna-seq-data-analysis. The VST values were median-centered for each sample, and means for all biological replicates were used for hierarchical clustering and calculating the *tau* tissue/organ specificity score, as described above.

### *PtMDs* Involved in Wood Biosynthesis

The AspWood high-spatial-resolution RNA-Seq dataset (Sundell et al., 2017) was used for analysis of expression of *PtMDs* during wood biosynthesis. The database provides VST expression values for four trees. Identity of wood developmental zones was based on the expression of marker genes (Sundell et al., 2017). A heatmap of *PtMD* expression in wood developmental zones was constructed for one representative tree (tree 1) using the AspWood server^15^.

### Co-expression Analysis

*PtMD* genes from the selected expression clusters were used as ‘Guide Genes’ to obtain co-expression networks for developing secondary tissues, using the AspWood program (see text footnote 14). The AspWood calculates co-expression networks utilizing mutual information and context likelihood of relatedness as explained by Sundell et al. (2017). The corresponding GraphML files were generated using the ExNet tool^16^ with a Z-score threshold of 5.0, and visualized using Cytoscape 3.4.0 (Shannon et al., 2003).

## Results and Discussion

### Identification of MD Proteins in *P. trichocarpa* and Their Classification

Searches of the *P. trichocarpa* and *A. thaliana* genomes for MD proteins resulted in the identification of 146 and 87 gene models, respectively (Supplementary Tables 1, 2). Previous analyses identified 62 *MD* genes in strawberry (Zhang et al., 2016), 74 in *A. thaliana* (Bellande et al., 2017; Sultana et al., 2020), and 84 in rice (Jing et al., 2020).

**FIGURE 1:**
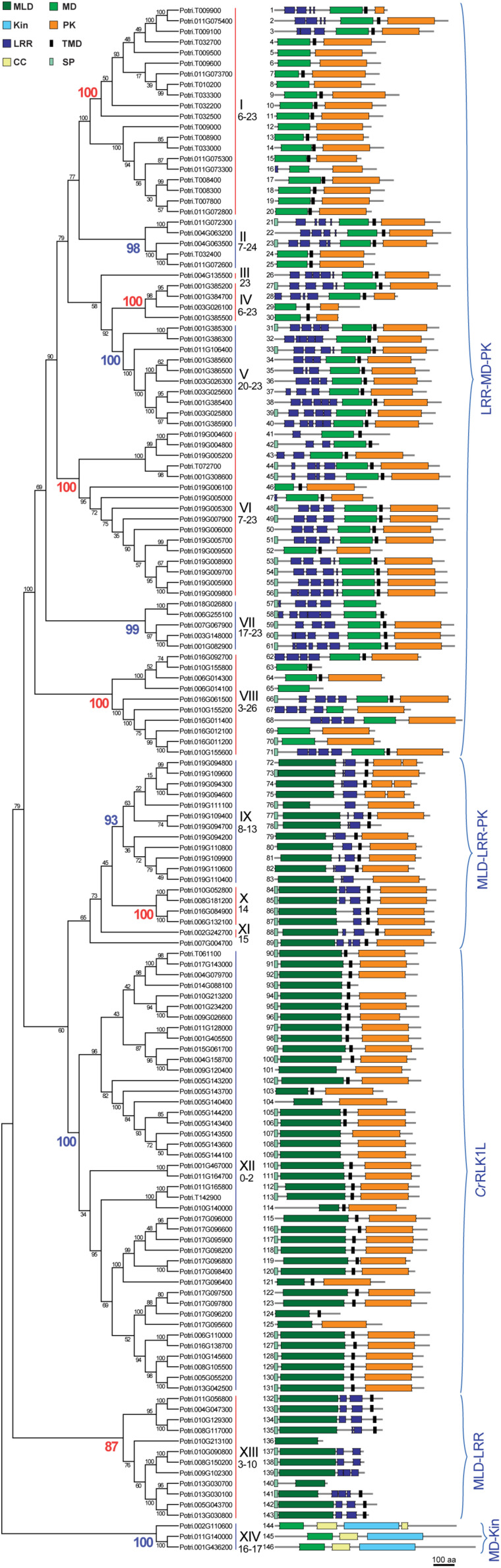
Phylogenetic tree of poplar malectin/malectin-like domain-containing proteins (*Pt*MDs) showing their domain structures. The tree was constructed from MUSCLE-aligned amino acid sequences using the neighbor-joining method in MEGA 7.0 with 1000 bootstrap replicates and bootstrap support is displayed beside the nodes as percentages. *Pt*MDs are identified by the number shown next to each protein structure. Domain abbreviations are: CC, coiled coil; Kin, kinesin; LRR, leucine-rich repeat; MD, malectin domain; MLD, malectin-like domain; PK, protein kinase; SP, signal peptide; TMD, transmembrane domain. Main clades are numbered with Roman numerals and their corresponding bootstrap values are colored in the phylogenetic tree. Numbers below the Roman numerals correspond to the number of introns observed within a clade. Five groups containing clades with similar protein domain structures are identified by the blue brackets: LRR-MD-PK (also known as poplar LRR-RLK XIII; Zan et al., 2013), MLD-LRR-PK (known as poplar LRR-RLK I; Zan et al., 2013), *Cr*RLK1L, MLD-LRR, and MD-Kin.

**FIGURE 2:**
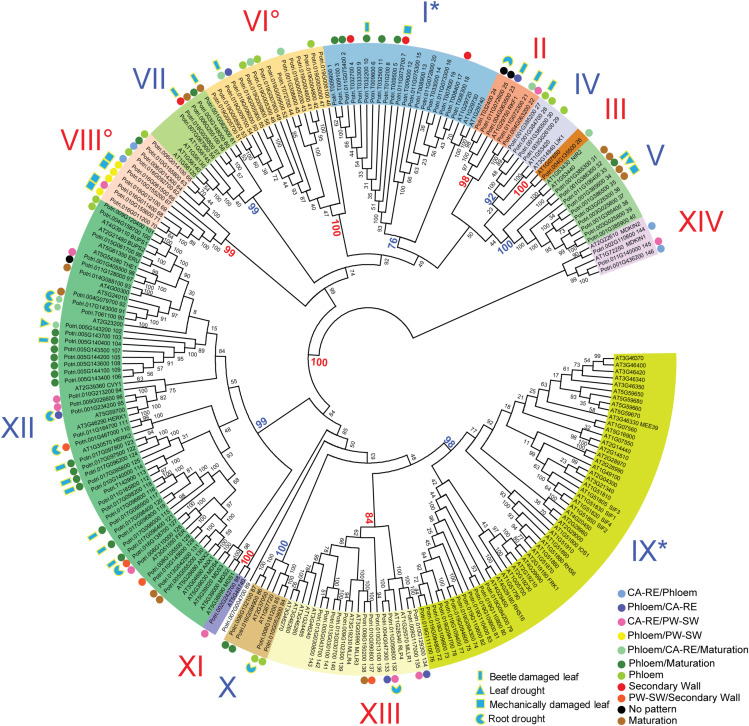
Phylogenetic tree of malectin/malectin-like domain-containing proteins in *P. trichocarpa* and *A. thaliana*. Each protein ID is followed by the name (*A. thaliana*) or *Pt*MD protein number (*P. trichocarpa*). The phylogenetic tree was constructed based on MUSCLE-aligned amino acid sequences using the neighbor-joining method in MEGA 7.0 using 1000 bootstrap replicates, and the bootstrap support is displayed in percentages. Main clades are numbered with Roman numerals, and their supporting bootstrap values are shown in color. Colored dots beside *PtMDs* identify genes expressed in secondary vascular tissues based on the AspWood (http://aspwood.popgenie.org/aspwood-v3.0/) database and showing maximum expression in different developmental zones as indicated by colors. CA-RE, cambium-radial expansion zone; PW-SW, primary to secondary wall transition zone. Blue shapes with yellow outlines show stress-related expression based on the aspen expression atlas available at http://popgenie.org. Degree symbols and asterisks beside Roman numerals indicate clades that are represented by only one species or are significantly expanded in one species (χ^2^-test, *P* ≤ 0.05), respectively.

The *P. trichocarpa* proteins identified were analyzed for sequence similarity using protein sequence alignment and phylogenetic analysis, revealing the presence of 12 clades supported by at least 87 % of bootstrap replicates, and three ungrouped sequences, two of which had orthologous sequences in *A. thaliana*, and were therefore considered to be two single member clades III and XI (Figures 1, 2). The sequences were numbered *PtMD1* to *PtMD146* according to their sequential appearance in the intraspecific phylogenetic tree (Figure 1). The predicted protein properties and probable subcellular localizations of *Pt*MD proteins are listed in Supplementary Table 1. The deduced sequence lengths ranged from 274 to 1192 amino acids, and isoelectric points (pIs) ranged from 4.55 to 9.49. Seventy-six out of the 146 *Pt*MD proteins had a signal peptide (SP) cleavage site. The SP was not found in any members of clades I and XIV. Thirteen of the *Pt*MDs were predicted to be soluble proteins, with the predicted localization of six of them being extracellular, six - including all members of clade XIV - being cytoplasmic and one being peroxisomal. Out of 133 membrane proteins, one was predicted to localize in the endoplasmic reticulum.

**FIGURE 3:**
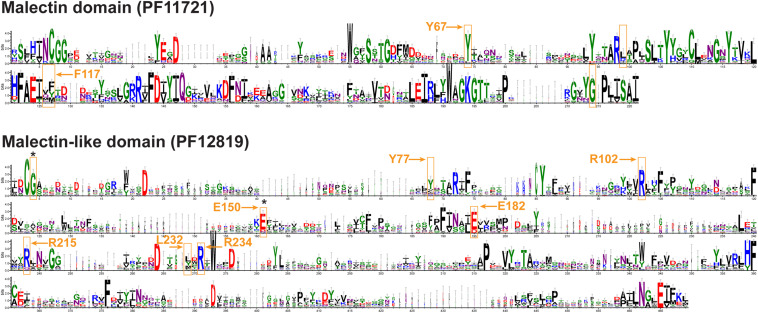
Conserved motifs in malectin (top) and malectin-like (bottom) domains of *P. trichocarpa*. The residues involved in interaction with ligands according to Schallus et al. (2008) and Moussu et al. (2018) are shown in orange boxes. The amino acids conserved in *P. trichocarpa* are marked by arrows. The mutated residues in the THE1 protein in the *the1* mutants studied (Hématy et al., 2007) are indicated by black asterisks. The logos were created based on *P. trichocarpa* MD/MLD amino acid sequence alignments by using the WebLogo 3.7.4 online tool (http://weblogo.threeplusone.com, Crooks et al., 2004). The conservation of amino acid residues is represented in bits and residues are colored according to chemical properties. The associated multiple sequence alignments are shown in Supplementary Figures 1, 2.

Domain analysis (Figure 1 and Supplementary Table 1) revealed the presence of two major groups, one with MD (clades I–VIII, and XIV) and the other with MLD (clades IX to XIII). There was relatively little conservation in the amino acid sequence between the two domains (Figure 3), and in many cases, the proteins having MLD were not classified as members of the CBM57 family (Supplementary Table 1; Kumar et al., 2019). Nevertheless, similarity between MD and each of the two sub-domains of MLD has previously been shown by comparisons of their 3D structures (Moussu et al., 2018). Among the conserved residues of MD, which were proposed to interact with diglucose in *Xenopus laevis* malectin (Y67, Y89, Y116, F117, and D186) (Schallus et al., 2008, 2010), only Y67 and F117 were conserved in poplar MD (Figure 3 and Supplementary Figure 1). In contrast, the residues proposed to interact with ligands in the MLD of ANX1, Y77, R102, E150, E182, R215, L232, and R234 (Moussu et al., 2018) were to a large extent conserved in poplar MLD (Figure 3 and Supplementary Figures 2A,B).

Clades I-XII had MD or MLD followed by a transmembrane helix preceding the protein kinase domain, in agreement with the typical topology of RLKs (Figure 1 and Supplementary Table 1). Protein kinase domains were frequently of the Tyr kinase type. Some MD proteins with a kinase domain had extracellular localization predicted for this domain (Supplementary Table 1). Clade V was the only one in which all kinase domains were predicted to be intracellular. Extracellular kinase domains were also predicted for the majority of G- and L-type lectin RLKs in poplar (Yang et al., 2016) but experimental validation of such predictions is currently lacking.

In all clades but XII and XIV, several LRR domains were found in tandem repeats. The LRR domain forms a horseshoe-like structure that functions in protein-protein or protein-ligand interactions (Bella et al., 2008). LRRs are known to occur in LRR-RLKs, receptor-like proteins (RLPs), resistance (R) proteins, LRR extensins (LRX), and other families (Wang et al., 2008; Draeger et al., 2015; Choi et al., 2016; Song et al., 2018). Poplar MD proteins had various types of LRR domains, most frequently LRR_4 and LRR_8 (Supplementary Table 1). The proteins from clade XIII had unique combinations of LRR domains; *Pt*MD134 and *Pt*MD135 had the sd00031 LRR domain, *Pt*MD141 had the LRR_1 domain, and *Pt*MD137, -138, and -139 had the LRRNT2 domain. The LRR domains found in the poplar MD family either preceded MD (clades I-VIII) or followed MLD (clades IX-XI and XIII) (Figure 1). Thus, the placement of LRR domains correlated with the presence of either MD or MLD. In clades with members containing LRRs, there were also several members devoid of any LRRs. This probably indicates domain loss due to unequal crossing over. A previous study on poplar LRR-RLKs (Zan et al., 2013) identified and classified some of the MD proteins studied here; MD clades I-VIII were previously classified as LRR-RLK group XIII, and MD clades IX-XI as LRR-RLK group I (Supplementary Table 1).

Clade XI and clade XII members exhibited an unusual TMD, CD12087, which is typical of epidermal growth factor receptors of animals where it functions in receptor dimerization (Mineev et al., 2010). Whether it can carry out such a function in plant MD proteins remains to be investigated.

Clade XIV domain structure and topology was unique in having a kinesin domain (Kin) along with an N-terminal MD (Figure 1). This clade has not been previously included in the surveys of *MD* genes in *A. thaliana* (Bellande et al., 2017) or rice (Jing et al., 2020). Recently, one of the *A. thaliana* clade XIV members, MDKIN2, was found to function in pollen and seed development (Galindo-Trigo et al., 2020). Orthologs of the clade XIV genes could be identified in many species of Viridiplantae, including moss, lower vascular plants, dicots and monocots.

Based on domain composition and domain order, poplar MD gene clades could be grouped into a higher order organization with five superclades characterized by the following domain patterns: 1) LRR-MD-PK (LRR-RLK group XIII, Zan et al., 2013), 2) MLD-LRR-PK (LRR-RLK group I, Zan et al., 2013), 3) MLD-PK (*Cr*RLK1L), 4) MLD-LRR (RLPs) and 5) MD-Kin (Figure 1).

### Chromosomal Distribution of *MD* Genes in *P. trichocarpa*

**FIGURE 4:**
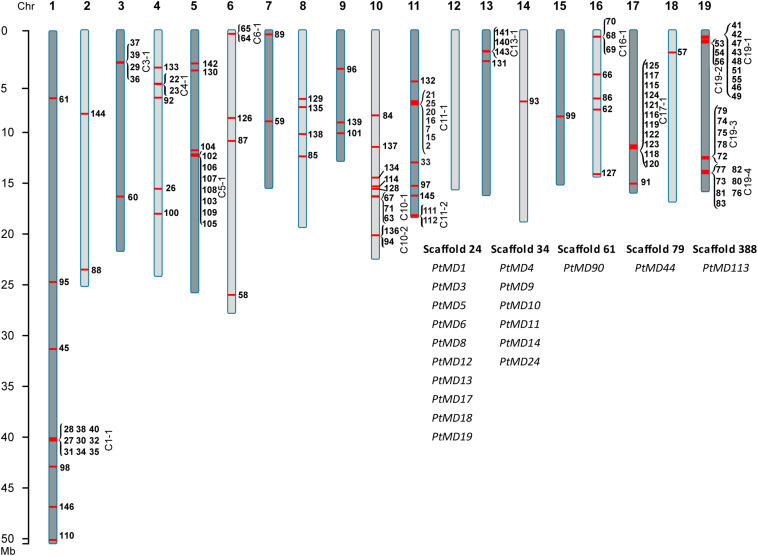
Chromosomal localization of the *PtMD* genes. The *PtMD* ID numbers are indicated beside chromosomes. Several *PtMDs* are found in clusters, shown by parentheses and labeled with a letter “C” followed by the chromosome number and the cluster number. Some *PtMDs* are on scaffolds, shown below the chromosomes.

127 out of the 146 poplar *MD* gene models were mapped to chromosomes, while 19 gene models were located on five different scaffolds (Figure 4). The majority of chromosomal genes (79) were present in clusters comprising between two and eleven genes (Figure 4 and Supplementary Table 3). Clusters were also present on the scaffolds. The clusters consisted of tandem repeats having the same or reverse orientations. This large number of tandem duplications strongly suggests that the main mechanism of MD family expansion in *P. trichocarpa* is via local gene duplication, rather than whole genome duplications. Gene multiplication at a given locus could occur via an unequal crossing over mechanism, which after multiple rounds would result in large numbers of tandemly repeated sequences. Such a mechanism was proposed as featuring particularly in various *LRR* gene families (Schaper and Anisimova, 2015) including *LRR-RLK* (Shiu and Bleecker, 2001; Zan et al., 2013; Zulawski et al., 2014; Zhang et al., 2016; Wang et al., 2019) and *R* genes (Choi et al., 2016). Indeed, 11 out of our16 clusters of *PtMD* genes had members with LRR domain(s) (Supplementary Table 3).

Tandem duplications allow rapid gene family expansion and the creation of novel alleles are thought to be particularly important for the co-evolution of *R* and *Avr* genes in hosts and their parasites (Holub, 2001; Choi et al., 2016). Partial duplications with omission of some domains form a key mechanism for neofunctionalization. Such a process apparently characterized the poplar MD family, since there were seven out of 16 clusters that included genes with LRR and kinase domains along with closely related members without LRR domains (Figure 4 and Supplementary Table 3).

### Analysis of Exon-Intron Structures of *PtMD* Genes

Exon-intron structure reflects the evolutionary history of genes; hence we analyzed the exon-intron organization of *PtMDs*. Although the majority of clades displayed very diverse numbers of introns (Supplementary Table 4 and Figure 1), the maximum number of introns for clades within a superclade was similar. The superclade LRR-MD-PK, comprising clades I-VIII, had genes with very large numbers of introns (maximum between 23 and 26); superclade MLD-LRR-PK (clades IX-XI) had at most 15 introns; superclade MLD-PK (clade XII or the *Cr*RLK1L group) contained genes with up to two, but typically without any introns; and superclades MLD-LRR (clade XIII) and MD-Kin (clade XIV) had at most 10 and 17 introns, respectively (Supplementary Table 4 and Figure 1). Lack, or low frequency, of introns in *Cr*RLK1L genes has also been observed in other species including strawberry, *Arabidopsis* and rice (Zhang et al., 2016; Bellande et al., 2017; Jing et al., 2020). Thus, the exon-intron organization of poplar *MD* genes supported their grouping into superclades, which represent ancestral diversification of plant *MD* genes.

### Comparison of *P. trichocarpa* and *A. thaliana* MD Proteins

The phylogenetic tree of MD proteins was generally consistent between *P. trichocarpa* and *A. thaliana* with bootstrap values of greater than 76 % for the main clades (Figure 2). Three exceptions were noted, however: one orphan protein *Pt*MD89, clade VI that included *Pt*MD41-*Pt*MD56, and clade VIII with *Pt*MD62-*Pt*MD71. These poplar genes apparently did not have orthologs in *A. thaliana*. To address a hypothesis that these genes represent tree-specific functions, we analyzed the MD gene families in other tree species with the whole genome data available (Supplementary Figure 3). Close homologs to *PtMD89* were found in other tree species including *Salix purpurea*, *Eucalyptus grandis*, *Theobroma cacao*, *Malus domestica*, *Prunus persica*, and *Citrus sinensis* (Supplementary Figure 3). Each of these species had only one putative *PtMD89* ortholog indicating that *PtMD89* function is conserved in angiosperm trees belonging to different families. Clades VI and VIII include tandemly repeated genes and were not well resolved by the phylogenetic analysis (Figure 4 and Supplementary Table 3). The clear homologs to *P. trichocarpa* tandemly replicated genes of clade VI were present in *Salix purpurea* although we cannot exclude that genes with less supported association to clade VI are present in *Prunus persica*, *Malus domestica*, *Theobroma cacao* and *Betula pendula* (Supplementary Figure 3). Clade VIII included only *P. trichocarpa* and *Salix purpurea* genes, but this clade had a weak bootstrap support (Supplementary Figure 3). Thus, the genes of clades VI and VIII had undergone recent tandem duplication in the *P. trichocarpa* lineage after its separation from that of *A. thaliana* that could be conserved in other members of Salicaceae. It is therefore possible that they represent specialized genes, such as *R* genes important for immunity, that co-evolved with poplar symbionts and/or pathogens of the Salicaceae family (Holub, 2001).

Besides identifying clades not represented in *Arabidopsis*, we found that the relative clade sizes (number of genes per clade relative to genome size) show some differences between the two species (Figure 2). Clade IX was expanded in *A. thaliana*, whereas clade I was expanded in *P. trichocarpa* (χ^2^-test at *P* ≤ 0.05). The phylogenetic analysis of *MD* genes including different tree species confirmed the expansion of clade IX in *A. thaliana* and clade I in *P. trichocarpa* (Supplementary Figure 3).

Domain composition and organization were consistent between *P. trichocarpa* and *A. thaliana* in clades present in both species (Supplementary Tables 1, 2). Previous studies in *A. thaliana* classified LRR-RLKs (Shiu and Bleecker, 2001) and assigned them putative receptor or co-receptor functions based on the sizes of ectodomains (Xi et al., 2019). Many MD proteins identified in the current study were among the previously classified LRR-RLKs (Supplementary Table 2). The group of clades I-VIII, except for clades VI and VIII, which were not represented in *A. thaliana*, have been classified as being of the LRR-VIII-2 class (Shiu and Bleecker, 2001). This group had large ectodomains including several LRR motifs followed by MD, TMD and internal kinase domains (LRR-MD-PK) (Supplementary Table 2 and Figure 1). *A. thaliana* proteins of clades IX-XI belong to class LRR-I (Shiu and Bleecker, 2001), having a large MLD ectodomain terminated with a short LRR repeat, TMD, and an internal protein kinase domain (MLD-LRR-PK), as was observed for poplar (Supplementary Table 2 and Figure 1). *A. thaliana* clade XII proteins correspond to the *Cr*RLK1L group, which is characterized by a large MLD ectodomain followed by TMD and the internal protein kinase domain, whereas clade XIII in *A. thaliana*, as in *P. trichocarpa*, was characterized by a large ectodomain including MLD and LRR domains. Such proteins are classified as RLPs (Wang et al., 2008).

Only four out of the 14 clades identified contained members that have been functionally analyzed in *A. thaliana*. In addition to clade XII (*Cr*RLK1L), which has been the most extensively studied, with members involved in CWI sensing, polar growth, fertilization, and immune responses (Franck et al., 2018), the members of clades IV, V, and IX have been functionally characterized. Clade IV includes LYSM RLK1-INTERACTING KINASE 1 (LIK1), an RLK interacting with the chitin receptor formed by the CERK1-LYSM RLK1 complex, which signals the presence of chitin and activates PTI (Le et al., 2014). Clade IX includes several RLKs involved in both immunity and development. For example, IMPAIRED OOMYCETE SUSCEPTIBILITY 1 (IOS1) acts as a co-receptor of flagellin, EF-Tu and chitin, interacting with FLS2, EFR, and CERK1, respectively (Yeh et al., 2016). STRESS INDUCED FACTORs 1-4 (SIF1, SIF2, SIF3 and SIF4) have been characterized as RLKs involved in biotic and abiotic stress responses (Yuan et al., 2018). SIF2 was found to interact with BAK1 and mediate PTI during pathogen attack. *FLG22-INDUCED RECEPTOR-LIKE KINASE 1* (*FRK1*) is known to be an early-induced PTI gene (Asai et al., 2002). *ROOT HAIR SPECIFIC 6* and *16* (*RHS6* and *RHS16*) were found to be specifically expressed in root hairs and *RHS16* overexpression dramatically altered root hair morphology, indicating an important function in root hair growth (Won et al., 2009), whereas MATERNAL EFFECT EMBRYO ARREST 39 (MEE39) was found to be essential for embryo development based on the mutant phenotype (Pagnussat et al., 2005).

### Expression of *PtMDs* in Different Organs of *Populus*

**FIGURE 5:**
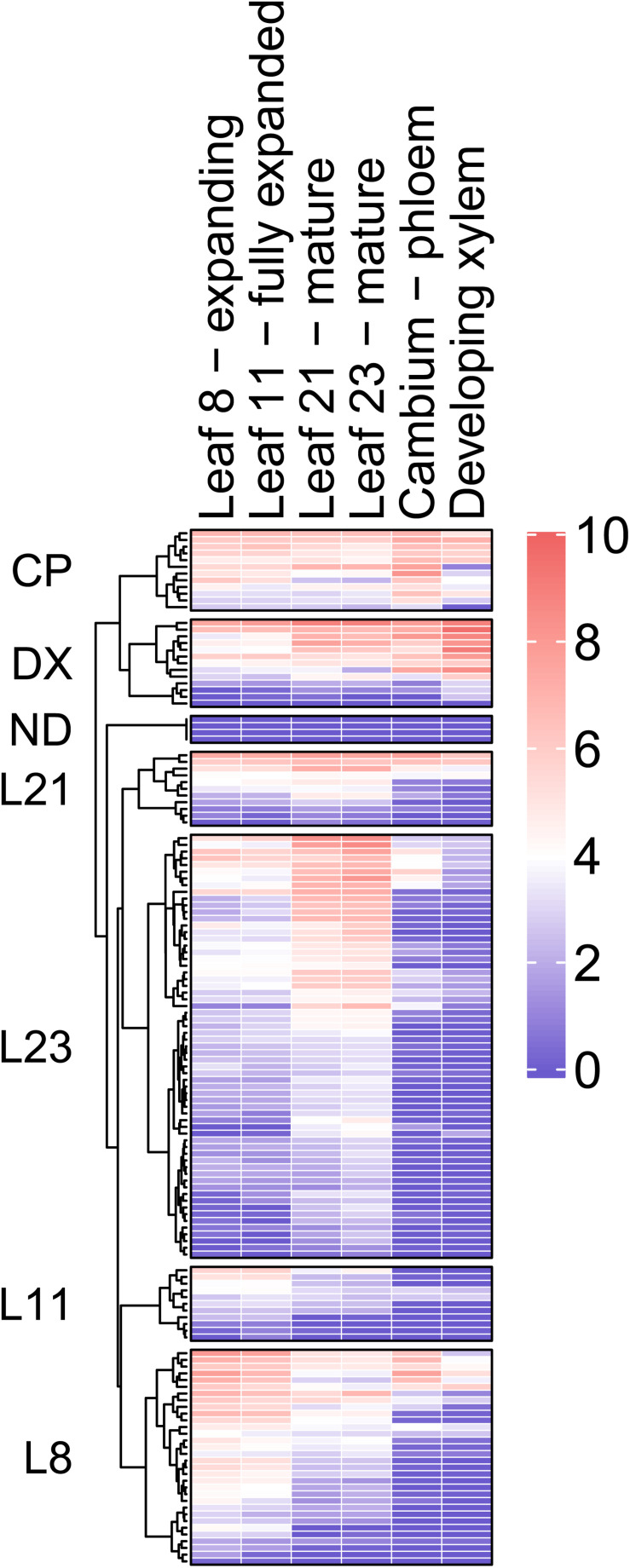
Heatmap of *PtMD* gene expression patterns in developing leaves and wood of hybrid aspen based on RNA sequencing. Details about the expression of each *PtMD* gene in the different tissues are listed in Supplementary Table 5. CP, cambium-phloem; DX, developing xylem; ND, not detected.

RNA sequencing strategy was adopted to examine the expression of 146 *PtMD* genes in developing leaves and wood tissues (Figure 5 and Supplementary Table 5). Datasets were centered by median expression in each sample and the variance-stabilized transformation (VST) expression values were clustered considering the tissue with maximum expression as a covariate. Majority of *PtMD* genes had the highest expression in the leaves, especially the fully mature ones (leaf 23). Relatively large number of *PtMD* genes was highly expressed in expanding leaves (leaf 8), and many of these genes were also highly expressed in the cambium. There were 12 and 13 *PtMD* genes with maximum expression in the cambium-phloem and developing xylem tissues, respectively. Majority of these genes showed generally high expression in leaves.

**FIGURE 6:**
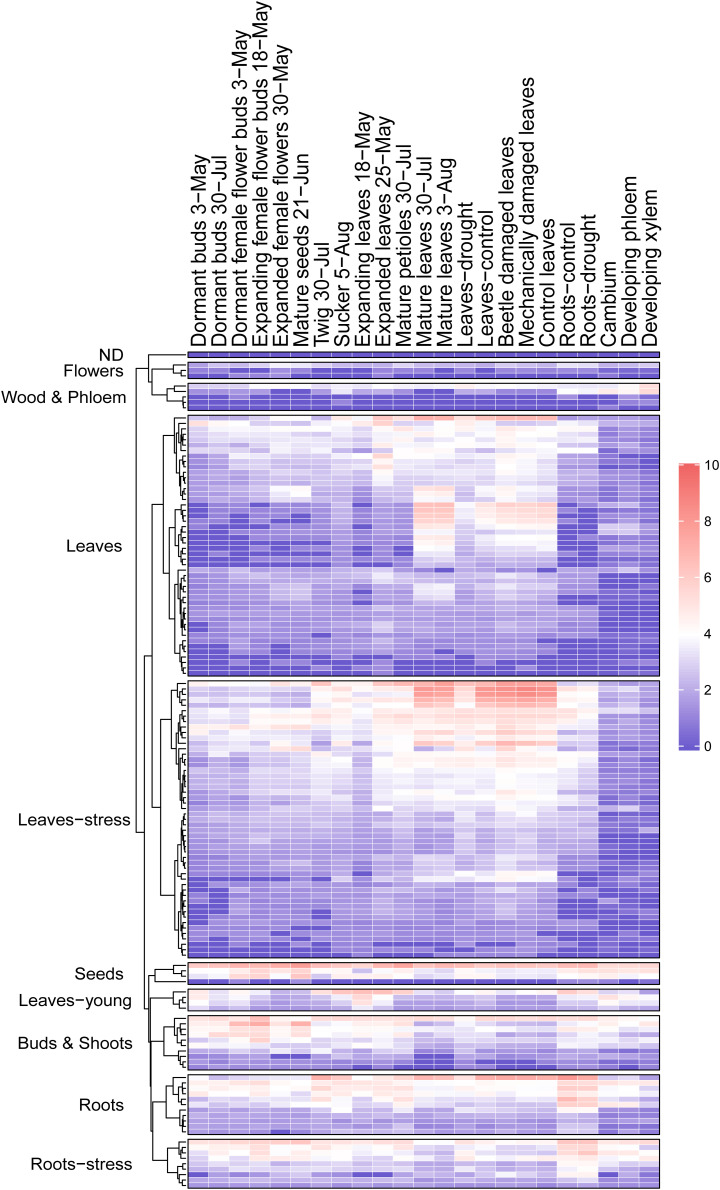
Heatmap of *PtMD* gene expression patterns in different organs of aspen. Data were retrieved from repositories described by Sundell et al. (2015) and Immanen et al. (2016), and normalized expression values are listed in Supplementary Table 6.

High expression in mature leaves suggests function in foliar defenses and homeostasis for majority of the *PtMD* genes. To investigate it, we also examined the RNA sequencing datasets available for different organs and tissues subjected to variety of stress and growth conditions in the greenhouse and in the field, and calculated VST expression values (Figure 6 and Supplementary Table 6) using the same approach as used for our leaf and wood developmental series. Considering all datasets examined (Figures 5, 6 and Supplementary Tables 5, 6), out of the 146 genes, 145 were expressed at least in one of the organs and tissues tested. Similar to our datasets, the majority of *PtMD* genes (99) showed maximum expression in leaves, especially the mature ones. Moreover, many of them (51) showed the highest expression in leaves exposed to abiotic/biotic stress, such as beetle, drought or mechanical damage. The genes highly expressed in mature and stress-exposed leaves usually exhibited high expression specificity as determined by the *tau* specificity score (Supplementary Table 6). Interestingly, genes belonging to the clades missing in *Arabidopsis* (VI and VIII) were found expressed, indicating that they are functional, and many of them showed a peak of expression in the mature and beetle or mechanically damaged leaves (Figure 2 and Supplementary Tables 5, 6) pointing to their involvement in stress responses. These observations support an important role of the leaf-expressed *PtMD* genes in foliar defense responses, some of which could be species-specific, as suggested by the phylogenetic analyses revealing differences in the presence and size of certain clades of *MD* genes (Figure 2 and Supplementary Figure 3) as well as by the expression analyses in different species. For example, almost all *MD* genes of strawberry (*Fragaria vesca*) were upregulated upon exposure to low temperature or drought stress (Zhang et al., 2016), whereas in rice (*Oryza sativa*), the expression levels of many *MD* genes greatly increased upon salt and drought stress, but not in response to low temperature (Jing et al., 2020).

Twenty *PtMD* genes were most highly expressed in roots of which nine showed highest expression in roots exposed to drought. Genes with maximal expression values detected in stressed organs were distributed among clades I, II, IV, V, VI, VII, VIII, X, XII, and XIII (Figure 2), suggesting stress response functions for these clades. Interestingly, no gene that was maximally expressed in stressed organs was found in clades III, IX, XI or XIV, suggesting their involvement in other types of signaling. Several *PtMD* genes were most highly expressed in the vegetative growing organs: young roots or leaves (Figures 5, 6 and Supplementary Tables 5, 6). Eight genes, all from clades XII and XIII, were most highly expressed in female flowers at various developmental stages, and four in mature seeds. The genes highly expressed in expanding female flower buds or in mature seeds were in many cases also highly expressed in developing secondary tissues, vascular cambium or developing secondary xylem and phloem (Figure 6 and Supplementary Table 6).

### *PtMDs* Involved in Wood Biosynthesis

**FIGURE 7:**
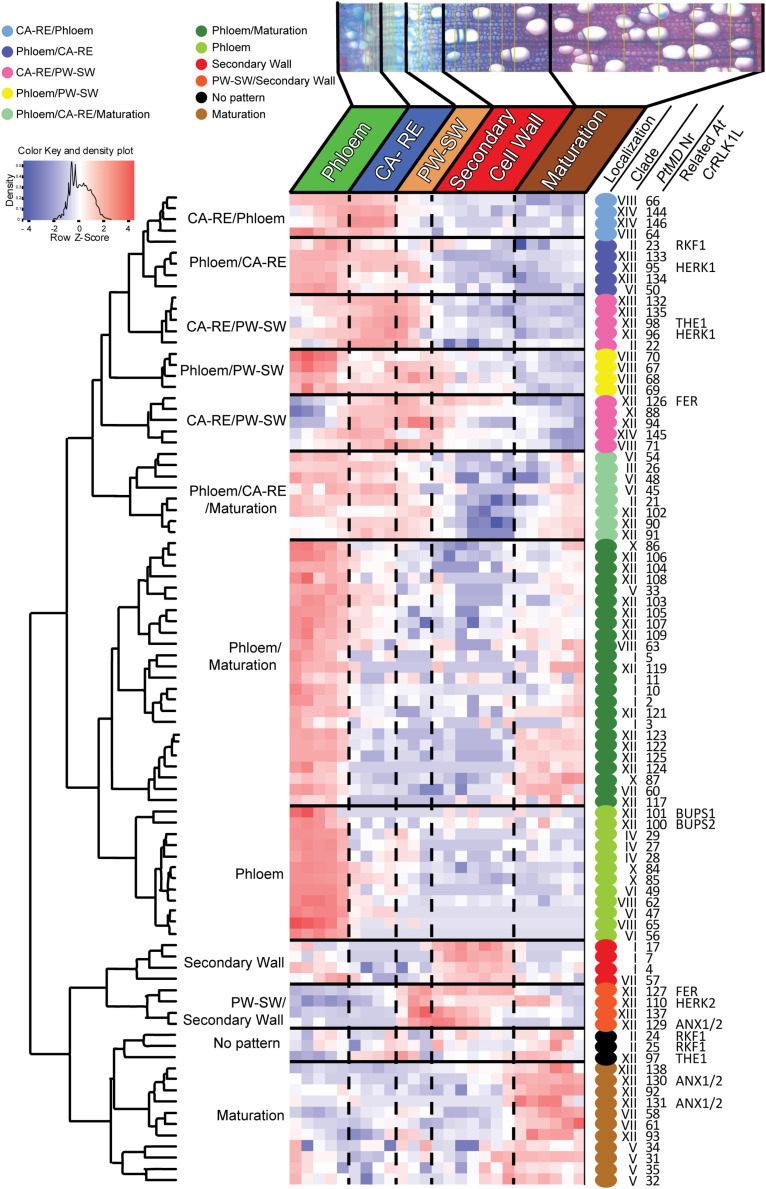
Heatmap of scaled *PtMD* expression patterns in developing secondary vascular tissues based on the AspWood database (http://aspwood.popgenie. org/aspwood-v3.0/). The majority of *PtMD* genes show maximum expression in the phloem and in the cambium-radial expansion zone (CA-RE). Smaller clusters of genes are expressed in the developing xylem including the secondary wall formation zone, the transition between the primary and secondary wall zone (PW-SW), or the maturation zone. Specific wood developmental stages are defined based on the patterns of expression of marker genes (Sundell et al., 2017). Colored dots beside *PtMDs* identify groups with maximum expression in different developmental zones.

Since many *PtMD* genes were found expressed in developing wood (Figure 5 and Supplementary Table 5), we wanted to determine at which wood developmental stage these genes are active. For that, we used the AspWood database (see text footnote 14), which provides data on high-spatial-resolution transcript abundance in developing secondary xylem and phloem tissues of aspen (Sundell et al., 2017). Only 89 *PtMD*s (61%) were found to be expressed in developing secondary vascular tissues (Supplementary Table 7), with the majority exhibiting distinct patterns of expression, clustering in ten expression groups (Supplementary Table 7 and Figure 7). This clustering indicates that certain sets of *PtMD*s have specific functions at certain stages of secondary vascular development. Some of the *PtMD* genes expressed in secondary vascular tissue also exhibited high expression under diverse stress conditions in leaves or roots (Supplementary Table 6 and Figure 2).

The largest group of *PtMD* genes (50) that were expressed in secondary vascular tissue showed a peak of expression in the phloem (Supplementary Table 7 and Figure 7). These genes were mostly from superclades LRR-MD-PK including many members of clade VI and VIII without orthologs in *Arabidopsis*, MLD-PK (*Cr*RLK1L), and MLD-LRR-PK. Cambium and radial expansion zones were the zones characterized by the greatest variety of *Pt*MD transcripts including members of superclades MD-Kin, LRR-MD-PK, MLD-LRR, and MLD-PK (*Cr*RLK1L). In contrast, *PtMD* genes having a peak of expression at the transition between primary and secondary wall deposition were mostly from the MLD-PK (*Cr*RLK1L) group. Intriguingly, the genes with maximum expression during secondary wall deposition were expressed at relatively low levels and many of them belonged to clade I of *Pt*MDs, which lacks LRR. *PtMD* genes with the highest expression in the maturation zone were mostly from clades V and XII.

### Networks of Xylogenesis-Related *PtMD* Genes

To find putative partners involved in signaling pathways together with the xylogenesis-related *PtMD* genes, we analyzed co-expression networks of *PtMD* genes identified as being expressed during xylogenesis. Ten *PtMD* genes forming two clusters with a peak of expression in the cambium-radial expansion zone and primary to secondary transition zone (CA-RE/PW-SW), and eight genes from clusters PW-SW/Secondary Wall and Secondary Wall (Figure 7 and Supplementary Table 7), representing, respectively, the early and main stages of secondary wall deposition were used as baits for network analyses.

**FIGURE 8:**
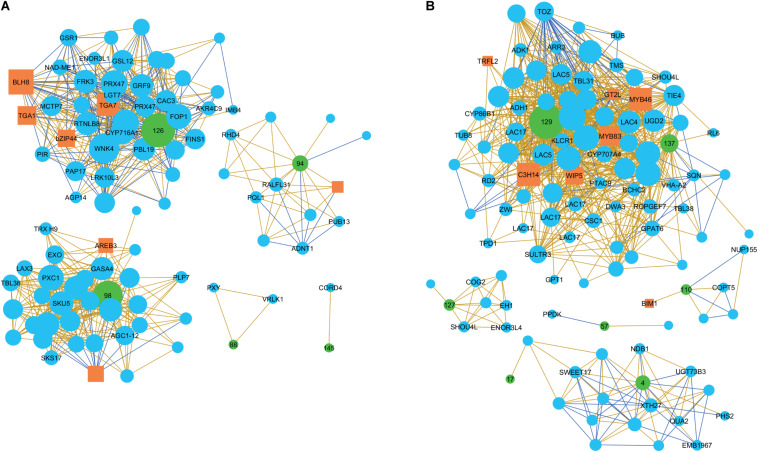
Co-expression networks of *PtMD*s in developing wood of aspen. The networks of genes expressed during the early **(A)** and main **(B)** stages of secondary wall deposition. The co-expressed genes were selected with threshold = 5 from the AspWood database (http://aspwood.popgenie.org/aspwood-v3.0/) and visualized in Cytoscape v 3.4.0. The transcription factors are marked by orange rectangles and the *PtMD* genes, used as baits, by green circles with *PtMD* ID numbers. *PtMD7, 22*, *71*, *9*6, *132* and *135* did not have any co-expressed genes (not shown). The corresponding *Arabidopsis* gene names are used in the figures. Positive and negative correlations are shown by beige and blue lines, respectively. The size of each marker corresponds to the number of correlations associated with it.

The baits for the CA-RE/PW-SW zones formed five separate networks (Figure 8A and Supplementary Table 8), the largest being that of *PtMD126* -one of the two poplar orthologs of *AtFER*. It included several candidates for functioning in signaling by phosphorylation relay and ROS, and for regulation of cell wall development. Apoplastic ROS in wood forming tissues could have a double role, in signaling and in regulation of lignin polymerization. Thus, the *PtMD126* network included a homolog of *PBS1-LIKE 19* (*AtPBL19*), encoding a RLCK of subfamily VII-4, which signals a response to chitin perceived by CHITIN ELICITOR RECEPTOR KINASE 1 (*At*CERK1) through a phosphorylation relay (Bi et al., 2018), and ROS production (Rao et al., 2018). Homologs of *AtTGA1* and *AtTGA7*, which encode basic leucine zipper transcription factors involved in oxidative stress-mediated responses to biotrophic and necrotrophic pathogens (reviewed by Gatz, 2013), were, respectively, positively and negatively correlated with *PtMD126* (Figure 8A and Table 1). The oxidation state of *At*TGA1 is regulated by a glutaredoxin, *At*ROXY19 (Li et al., 2019), the homolog of which has been found to respond to altered secondary wall xylan in aspen (Ratke et al., 2018), suggesting that the *PtMD126* network might include candidates for sensing secondary wall integrity. *At*TGA1 interacts with the BLADE-ON-PETIOLE 1 and 2 (*At*BOP1/2) transcription factors (Wang et al., 2019), which are known to regulate xylem fiber differentiation (Liebsch et al., 2014). Moreover, the network includes a homolog of the BEL1-LIKE HOMEODOMAIN 8 (*At*BLH8*)* transcription factor, which controls expression of BOP1 (Khan et al., 2015; Figure 8A and Table 1). The network also includes a homolog of the gene encoding GROWTH-REGULATING FACTOR 9 (*At*GRF9), a 14-3-3 protein that regulates developmental programs and stress signaling by binding phosphoproteins and regulating their activities (Mayfield et al., 2007; Liu et al., 2014; Omidbakhshfard et al., 2018). The presence of a homolog of IMPORTIN-BETA 4 (*At*IMB4), which is required to transport GRF-INTERACTING FACTOR 1 (*At*GIF1) to the nucleus (Liu et al., 2019; Figure 8A and Table 1) further supports the involvement of *GRF*/*14-3-3* genes in the *PtMD126* network.

**TABLE 1:** Genes co-regulated with poplar *MD* genes expressed during secondary wall formation that were discussed in the text.

				Baits								
First neighbors	Poplar name	Best BLAST AGI codes	Ath-names	MD126 (Potri.006G110000)	MD94 (Potri.010G213200)	MD98 (Potri.001G405500)	MD88 (Potri.002G242700)	MD129 (Potri.008G105500)	MD137 (Potri.010G090800)	MD110 (Potri.001G467000)	Ath short description	Pathway/process
Potri.014G052700		AT5G47070	PBL19	+							PBS1-LIKE 19 - a RLCK phosphorylating MAPKKK5 and MEKK1 in response to chitin	PAMP, ROS, P, JA, and BR signaling, BOP1/2
Potri.002G090700		AT5G65210	TGA1	+							TGA-BINDING 1 - a bZIP TF, a redox-controlled regulator of SAR and development	PAMP, ROS, P, JA, and BR signaling, BOP1/2
Potri.005G170500		AT1G77920	TGA7	−							TGA-BINDING 7 - a bZIP TF, a redox-controlled regulator of SAR and development	PAMP, ROS, P, JA, and BR signaling, BOP1/2
Potri.004G213300		AT2G27990	BLH8	+							BEL1-like TF, regulating BOP1 and integrating stress signaling via JA	PAMP, ROS, P, JA, and BR signaling, BOP1/2
Potri.001G392200		AT2G42590	GRF9	+							GROWTH-REGULATING FACTOR 9, 14-3-3 gene. Binds Ca^2 +^ and regulates development.	Ca^2+^and P signaling and regulation
Potri.010G169800		AT4G27640	IMB4	+							IMPORTIN-BETA 4 transporting GRF-interacting factor 1 (GIF1) to nucleus	Ca^2+^and P signaling and regulation
Potri.009G029600		AT3G46510	PUB13		+						PLANT U-BOX 13, an E3 ubiquitin ligase involved in ubiquitination of receptor FLS2.	PAMP signaling
Potri.005G100500		AT3G51460	RHD4		+						ROOT HAIR DEFECTIVE4, a phosphatidylinositol-4-P phosphatase required by root hairs	P signaling and regulation
Potri.015G108700		AT5G61820			+						Stress up-regulated Nod 19 protein;	Ca^2+^and P signaling and regulation
Potri.017G059500		AT4G13950	RALFL31		+						RAPID ALKALINIZATION FACTOR LIKE 31 - peptide hormone	*Cr*RLK1L -mediated signaling
Potri.005G174000		AT1G77690	LAX3			+					Auxin influx carrier LAX3 (Like Aux1)	Auxin signaling
Potri.010G236200		AT3G44610	AGC1-12			+					Kinase involved in phototropism and gravitropism. Phosphorylates PIN1	Auxin signaling
Potri.017G083000		AT5G15230	GASA4			+					Encodes GA-regulated protein GASA4. Promotes GA responses and exhibits redox activity.	GA signaling
Potri.006G117200		AT2G36570	PXC1			+					Leucine-rich repeat protein kinase family protein	Xylogenesis and SW formation
Potri.001G057800		AT1G67310				−					Calmodulin-binding TF	Ca^2+^-related signaling
Potri.001G126100		AT5G61480	PXY				+				PHLOEM INTERCALATED WITH XYLEM -a LRR-RLK, receptor of TDIF regulating xylem cell fate	Xylogenesis and SW formation
Potri.006G114400		AT1G79620	VRLK1				+				VASCULAR-RELATED RLK1 - a LRR kinase regulating onset of secondary cell wall thickening.	Xylogenesis and SW formation
Potri.009G053900	MYB021	AT5G12870	MYB46					+			Master secondary wall TF MYB46	Xylogenesis and SW formation
Potri.001G018900		AT1G51220	WIP5					+	+		WIP domain 5. Target of WRKY53, involved cell fate determination in response to auxin via MP.	Auxin signaling
Potri.008G105600		AT4G24972	TPD1					+			TAPETUM DETERMINANT 1, peptide hormone perceived by EMS1-SERK1	P signaling and regulation
Potri.001G267300	MYB3	AT3G08500	MYB83					+			Master secondary wall TF MYB83	Xylogenesis and SW formation
Potri.016G104400		AT5G02010	ROPGEF7						+		ROP (RHO OF PLANTS) GUANINE NUCLEOTIDE EXCHANGE FACTOR 7	*Cr*RLK1L -mediated signaling
Potri.005G135500		AT2G15790	SQN						−		SQUINT - homolog of cyclophilin 40, involved in miRNA regulation	miRNA regulation
Potri.004G005900		AT4G22120	CSC1						+		CALCIUM PERMEABLE STRESS-GATED CATION CHANNEL 1- stretch activated cation channel.	Ca^2+^-related signaling
Potri.005G051700		AT5G28300	GT2L					−			GT-2LIKE PROTEIN - a CaM-binding protein involved in cold stress signaling	Transcriptional regulation
Potri.001G295100		AT3G19590	BUB3.1					−			BUDDING UNINHIBITED BY BENZYMIDAZOL 3.1. - spindle assembly.	Cell division
Potri.001G394200		AT4G20010	PTAC9					−			PLASTID TRANSCRIPTIONALLY ACTIVE 9- a single-stranded DNA binding protein.	Cell division
Potri.013G079600		AT5G16750	TOZ					−			TORMOZ -rRNA processing required for cell division	Cell division
Potri.015G048000		AT5G08130	BIM1							+	BES1-INTERACTING MYC-LIKE 1-a BHLH TF involved in brassinosteroid signaling	BR signaling

*All genes from network analyses are listed in Supplementary Tables 8, 9.*

A separate large network was formed by neighbors of *PtMD98* -one of the two poplar orthologs of *AtTHE1* (Figure 8A, Table 1 and Supplementary Table 8). This network comprised genes related to hormonal signaling by IAA and GA, and to the regulation of xylogenesis. One example is a homolog of *AtLAX3*, which encodes an auxin influx carrier (Swarup et al., 2008). Another is a homolog of *AtAGC1-12*, encoding a kinase phosphorylating the auxin efflux carrier *At*PIN1 (Haga et al., 2018). We have also identified a homolog of *AtGASA4* involved in GA responses and redox regulation (Rubinovich and Weiss, 2010). It is noteworthy that GA responses and *GASA* genes were also found to be upregulated in response to a secondary wall xylan defect in aspen (Ratke et al., 2018). Moreover, the co-expression network included an LRR-RLK homologous to *At*PXY-CORRELATED 1 (*At*PXC1), which is required for secondary wall deposition (Wang et al., 2013).

The network of *PtMD94* -the clade XII member related to *AtHERK1* and *AtCVY1* -included a homolog of *AtRALFL31* (Figure 8A, Table 1 and Supplementary Table 8). *RALF* genes encode hormone peptides that signal developmental processes and stress responses by interacting with *Cr*RLK1Ls. *At*RALFL31 belongs to subfamily IIIA which includes as yet uncharacterized members, but both *At*RALFL31 and Potri.017G059500 have the conserved YISY motif essential for interaction with *At*FER (Campbell and Turner, 2017). Thus, *Potri.017G059500* could potentially encode a peptide hormone recognized by *Pt*MD94. The *PtMD94* network also included other candidates for signaling. For example, there was a homolog of *PLANT U-BOX 13* (*AtPUB13)*, which encodes an E3 ligase involved in signal-activated ubiquitination and subsequent degradation of different receptors including ABA INSENSITIVE 1 (*At*ABI1) (Kong et al., 2015), BRASSINOSTEROID INSENSITIVE 1 (*At*BRI1) (Zhou et al., 2018), LYSM-CONTAINING RECEPTOR-LIKE KINASE 5 (*At*LYK5) (Liao et al., 2017), and FLAGELLIN-SENSITIVE 2 (*At*FLS2) (Liu et al., 2012; Antignani et al., 2015). The ubiquitination of flg22-bound *At*FLS2 by *At*PUB13 depends on its interactor protein RAB GTPASE HOMOLOG A 4B (*At*RABA4B) (Antignani et al., 2015). Interestingly, a homolog to another *PtMD94* network member encodes ROOT HAIR DEFECTIVE 4 (*At*RHD4) which mediates polar localization of *At*RABA4B (Thole et al., 2008). Consequently, it seems likely that these *PtMD94* network members are indeed functionally linked within the same network.

Two other *PtMD* genes expressed during early secondary wall biosynthesis, *PtMD88* and *PtMD145*, formed small networks, which included important regulatory genes in xylem cell differentiation (Figure 8A, Table 1 and Supplementary Table 8). One of them was the homolog of the master spatial regulator of vascular differentiation, *PHLOEM INTERCALATED WITH XYLEM* (*AtPXY*), encoding an LRR-RLK that promotes cell division in the cambium upon binding the small CLE peptide *At*TDIF, which is essential for xylem differentiation (Fisher and Turner, 2007). The other was a homolog of *VASCULAR-RELATED RECEPTOR-LIKE KINASE 1* (*AtVRLK1*) (Huang et al., 2018) which is probably responsible for the switch between xylem cell expansion and secondary wall deposition.

The late secondary wall-expressed baits formed five networks (Figure 8B, Table 1 and Supplementary Table 9). The largest of these was associated with two *PtMD* genes, *PtMD129*, a clade XII member related to *AtANX1* and *AtANX2*, and *PtMD137*, which encodes an LRR-RLK, from clade XIII. Orthologs of key signaling-related genes were included within this network. One of them was *CALCIUM PERMEABLE STRESS-GATED CATION CHANNEL 1 (AtCSC1*). Stretch-activated Ca^2+^ channels have been predicted to be important players in CWI (Engelsdorf and Hamann, 2014). *At*CSC1 belongs to a newly characterized family of stretch-activated Ca^2+^ channels conserved in eukaryotes (Hou et al., 2014; Liu et al., 2018). In addition, we found *Potri.015G108700/AT5G61820*, encoding an uncharacterized NOD19-like protein, which has been implicated in responses to cold stress downstream of mechanosensitive Ca^2+^ channels (Mori et al., 2018). The aspen homolog of *AtCSC1* is thus a promising candidate for a secondary wall damage sensor. Another important signaling-related homolog is *TAPETUM DETERMINANT 1* (*AtTPD1*), which encodes a small peptide hormone that is recognized by an RLK complex consisting of *At*EMS1 and *At*SERK1/2 to activate transcription factors of the BES1 family (Chen et al., 2019). Moreover, a homolog of *Arabidopsis ROP (RHO OF PLANTS) GUANINE NUCLEOTIDE EXCHANGE FACTOR 7* (*AtROPGEF7*) was among the hits for *PtMD137*. *At*ROPGEF7 interacts with the kinase domain of *At*FER, mediating downstream NADPH oxidase-dependent ROS signaling which is needed for polarized cell growth (Duan et al., 2010). Finally, we identified a homolog of *Arabidopsis GT-2LIKE PROTEIN* (*AtGT2L*), which encodes a Ca^2+^-dependent calmodulin (CaM)-binding trihelix transcription factor involved in plant abiotic stress signaling (Xi et al., 2012). In addition to signaling-related genes, the *PtMD129-PtMD137* network included some key cell fate regulator proteins (Figure 8B, Table 1 and Supplementary Table 9). One of these was the homolog of *WIP DOMAIN PROTEIN 5* (*AtWIP5*), which encodes a zinc-finger protein involved in root patterning downstream of auxin (Crawford et al., 2015) and ROS signaling (Miao et al., 2004). Another was a homolog of *Arabidopsis SQUINT* (*AtSQN*), which encodes a cyclophilin 40-like protein that promotes the accumulation of miRNAs miR156 and miR172, targeting master regulatory genes in organ development (Smith et al., 2009; Prunet et al., 2015). The network also included orthologs of two genes encoding master transcriptional regulators, *At*MYB46 and *At*MYB83, which activate the secondary wall program (Zhong and Ye, 2012). Both these genes showed positive correlation with *PtMD129*. In contrast, orthologs of three genes with roles in cell division were negatively correlated with *PtMD129* (Table 1). This supports the hypothesis that secondary wall integrity signaling results in coordination between cell division and secondary wall formation activities in developing wood (Ratke et al., 2018).

The network for *PtMD110*, which together with *PtMD111* forms a pair orthologous to *AtHERK2*, included a homolog of the *Arabidopsis* gene encoding the transcription factor BES1-INTERACTING MYC-LIKE1 (*At*BIM1) (Figure 8B, Table 1 and Supplementary Table 9), which mediates brassinosteroid signaling (Chandler et al., 2009). Several other genes discussed above can be linked to BR-dependent or BES-related BR-independent signaling (Table 1). Intriguingly, a secondary wall xylan defect induced transcriptomic changes suggesting stimulation of BR signaling in aspen (Ratke et al., 2018), supporting the involvement of the *AtBIM1* homolog in sensing secondary wall integrity.

## Conclusion

Malectin and malectin-like domains (MD/MLD) are lectin-like motifs found in proteins (MD proteins) of pro- and eukaryotes; they are particularly abundant in plants, where they carry out essential signaling functions in defense and development (Bellande et al., 2017; Franck et al., 2018). This has been shown by studies on *MD* genes from herbaceous plants such as *Arabidopsis* (Bellande et al., 2017; Sultana et al., 2020), strawberry (Zhang et al., 2016) and rice (Jing et al., 2020). However, no such comprehensive study has been available for *MD* genes in trees. Here we carried out a census of *MD* genes in the model woody species *P. trichocarpa* (Supplementary Table 1) and expanded the set for *A. thaliana* (Supplementary Table 2).

In total, 146 *MD* genes were found in *P. trichocarpa* and they were assigned to fourteen clades based on sequence similarity, and to five superclades based on predicted protein domain organization and intron-exon structures (Figures 1, 2). The variety of MD protein structures reflects their range of different functions in plants.

Additional genome-wide analysis by using available sequence data from different woody species revealed, that certain *MD* genes appeared to be specific either to trees or to the *Populus* lineage and absent from *Arabidopsis* (Supplementary Figure 3). The prevalence of tandem duplications within the *MD* gene family, which apparently led to family expansion, may have created conditions conducive to gene neofunctionalization and rapid evolution (Schaper and Anisimova, 2015; Choi et al., 2016).

The majority of the poplar *MD* genes were found to be highly expressed in mature leaves, particularly those subjected to biotic and abiotic stress conditions (Figure 6), supporting their role in stress signaling. Detailed analysis of expression in wood forming tissues revealed subsets upregulated in xylem cells during secondary wall deposition (Figure 7). These genes, not unexpectedly, include candidates for the sensing of cell wall integrity. We identified their co-expression networks revealing potential molecular pathways in which these *MD* genes might participate to ensure the coordination of secondary wall formation (Table 1).

The current study provides an extensive analysis of *Populus MD* genes and opens the possibility to better understand their role in essential physiological pathways related to stress signaling and the regulation of wood formation in trees.

## Data Availability Statement

The datasets presented in this study can be found in online repositories. The names of the repository/repositories and accession number(s) can be found in the article/Supplementary Material.

## Author Contributions

VK identified *MD* genes and their chromosomal clustering, and wrote the first draft. VK and FB identified protein domains and the main clades of *MD* genes. VK and SK analyzed exon-intron structures. ED analyzed gene expression in leaves and wood. VK and ED analyzed *in silico* gene expression in different organs. ED, JU, and VK analyzed conserved regions in MD and MLD of poplar. VK and FB analyzed co-expression networks. VK and CM analyzed the phylogeny across the tree species. EM conceived and coordinated the project, and finalized the manuscript with contributions from all authors. All authors contributed to the article and approved the submitted version.

## Conflict of Interest

The authors declare that the research was conducted in the absence of any commercial or financial relationships that could be construed as a potential conflict of interest.

## Footnotes

1 http://www.cazy.org/

2 http://popgenie.org

3 https://pfam.xfam.org

4 http://www.arabidopsis.org/

5 http://cdvist.zhulinlab.org

6 https://web.expasy.org/protparam/

7 www.cbs.dtu.dk/services/SignalP/

8 http://www.cbs.dtu.dk/services/DeepLoc/

9 http://popgenie.org/gbrowse

10 http://popgenie.org/chromosome-diagram

11 http://phylogeny.lirmm.fr/phylo_cgi/index.cgi

12 https://genome.jgi.doe.gov/portal/pages/dynamicOrganismDownload.jsf?organism=Phytozome

13 www.bioinformatics.babraham.ac.uk/projects/fastqc/

14 https://www.ebi.ac.uk/ena/browser/view/PRJEB41170

15 http://aspwood.popgenie.org/aspwood-v3.0/

16 http://popgenie.org/exnet
