# Supplementary material for: Genome-Wide Identification of Populus Malectin/Malectin-Like Domain-Containing Proteins and Expression Analyses Reveal Novel Candidates for Signaling and Regulation of Wood Development
Source: Front Plant Sci. 2020 Dec 22;11:588846. doi: 10.3389/fpls.2020.588846 (PMC7783096; doi:10.3389/fpls.2020.588846)
Supplement: Supplementary file 6 [file Presentation_1.PPTX]

## Slide 1
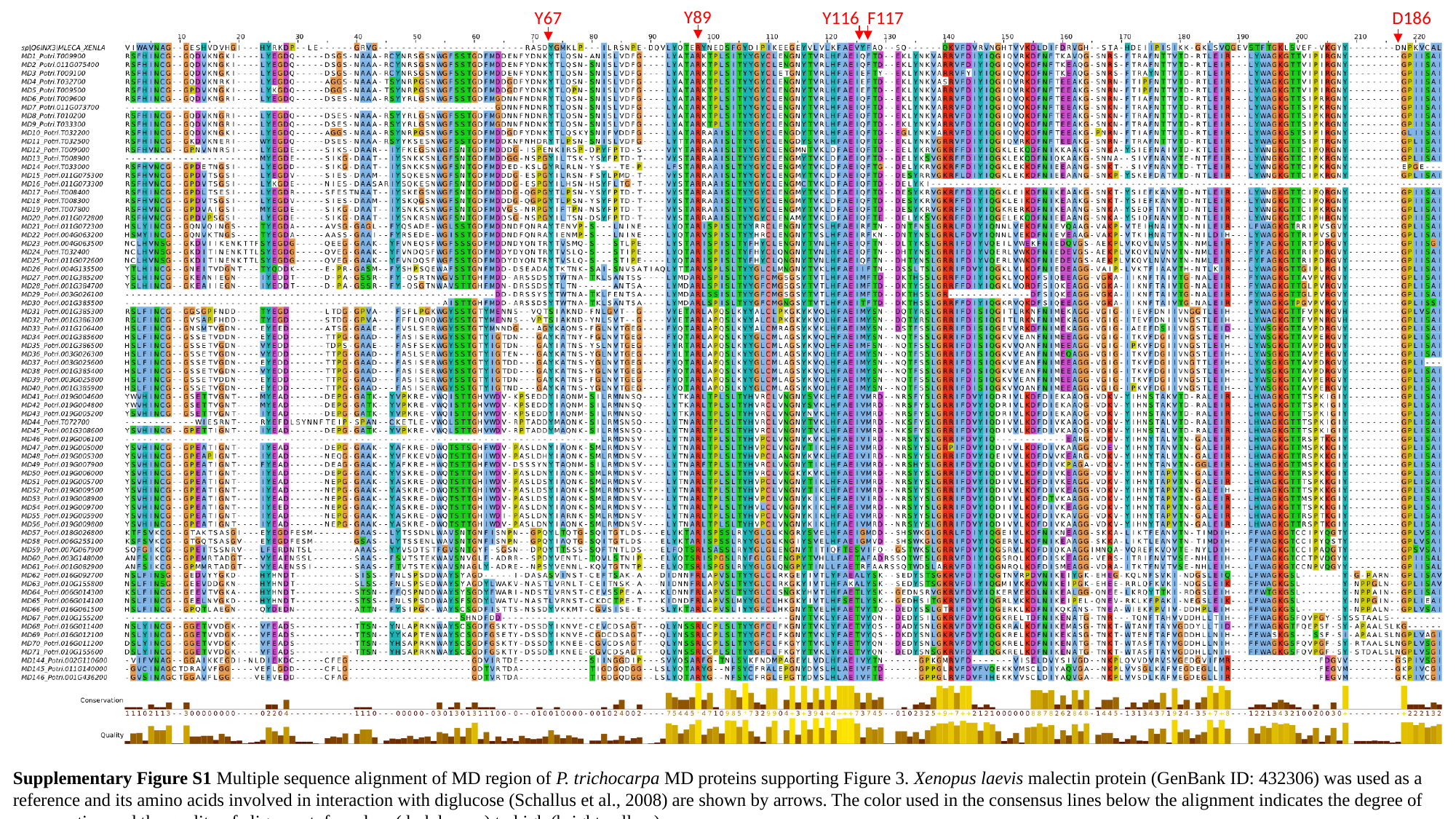

Y89
D186
Y67
Y116 F117
Supplementary Figure S1 Multiple sequence alignment of MD region of P. trichocarpa MD proteins supporting Figure 3. Xenopus laevis malectin protein (GenBank ID: 432306) was used as a reference and its amino acids involved in interaction with diglucose (Schallus et al., 2008) are shown by arrows. The color used in the consensus lines below the alignment indicates the degree of conservation and the quality of alignment, from low (dark brown) to high (bright yellow).
