# Supplementary material for: Genome-Wide Identification of Populus Malectin/Malectin-Like Domain-Containing Proteins and Expression Analyses Reveal Novel Candidates for Signaling and Regulation of Wood Development
Source: Front Plant Sci. 2020 Dec 22;11:588846. doi: 10.3389/fpls.2020.588846 (PMC7783096; doi:10.3389/fpls.2020.588846)
Supplement: Supplementary file 7 [file Presentation_2.PPTX]

## Slide 1
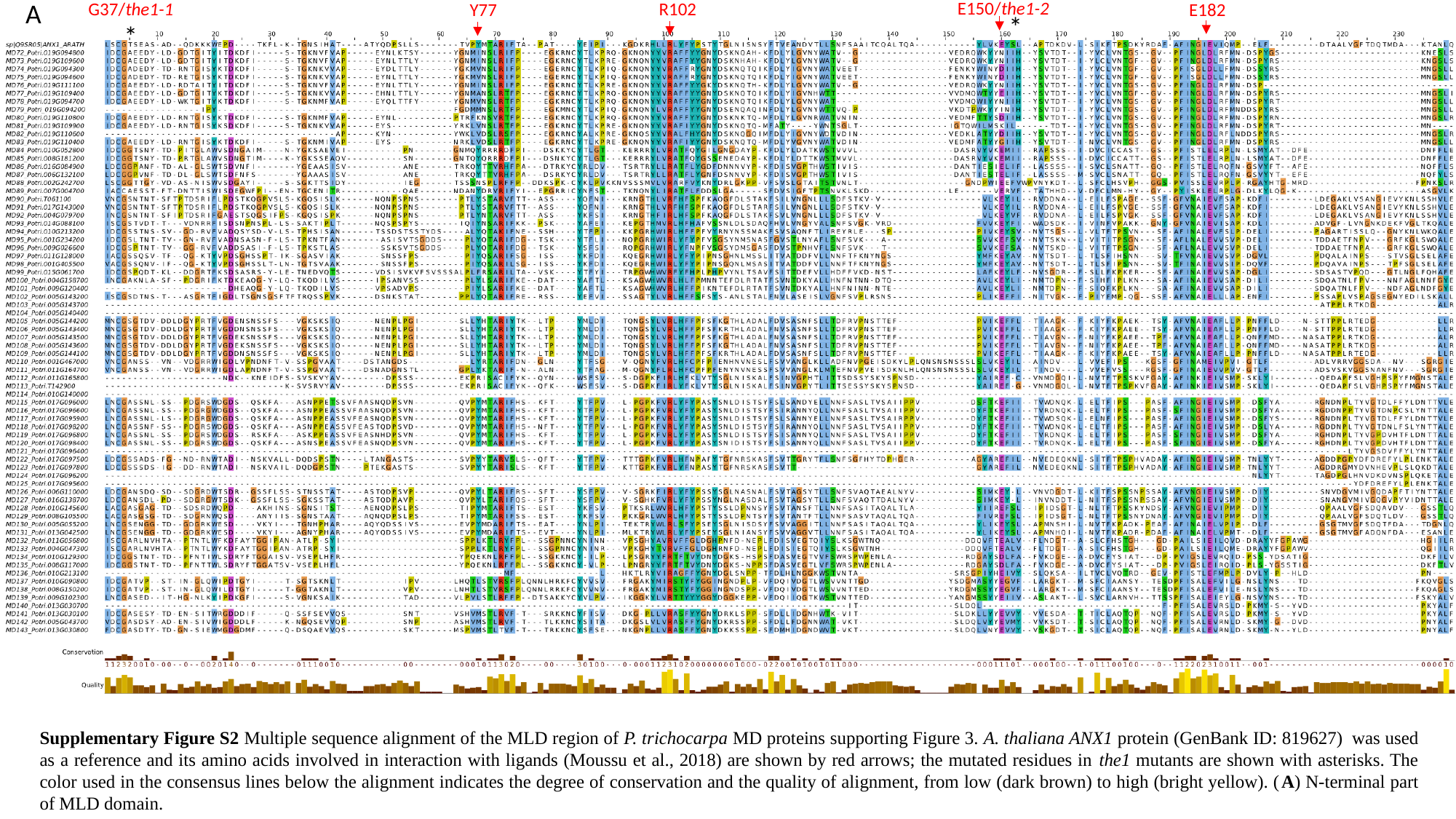

A
E150/the1-2
G37/the1-1
R102
E182
Y77
*
*
Supplementary Figure S2 Multiple sequence alignment of the MLD region of P. trichocarpa MD proteins supporting Figure 3. A. thaliana ANX1 protein (GenBank ID: 819627) was used as a reference and its amino acids involved in interaction with ligands (Moussu et al., 2018) are shown by red arrows; the mutated residues in the1 mutants are shown with asterisks. The color used in the consensus lines below the alignment indicates the degree of conservation and the quality of alignment, from low (dark brown) to high (bright yellow). (A) N-terminal part of MLD domain.

## Slide 2
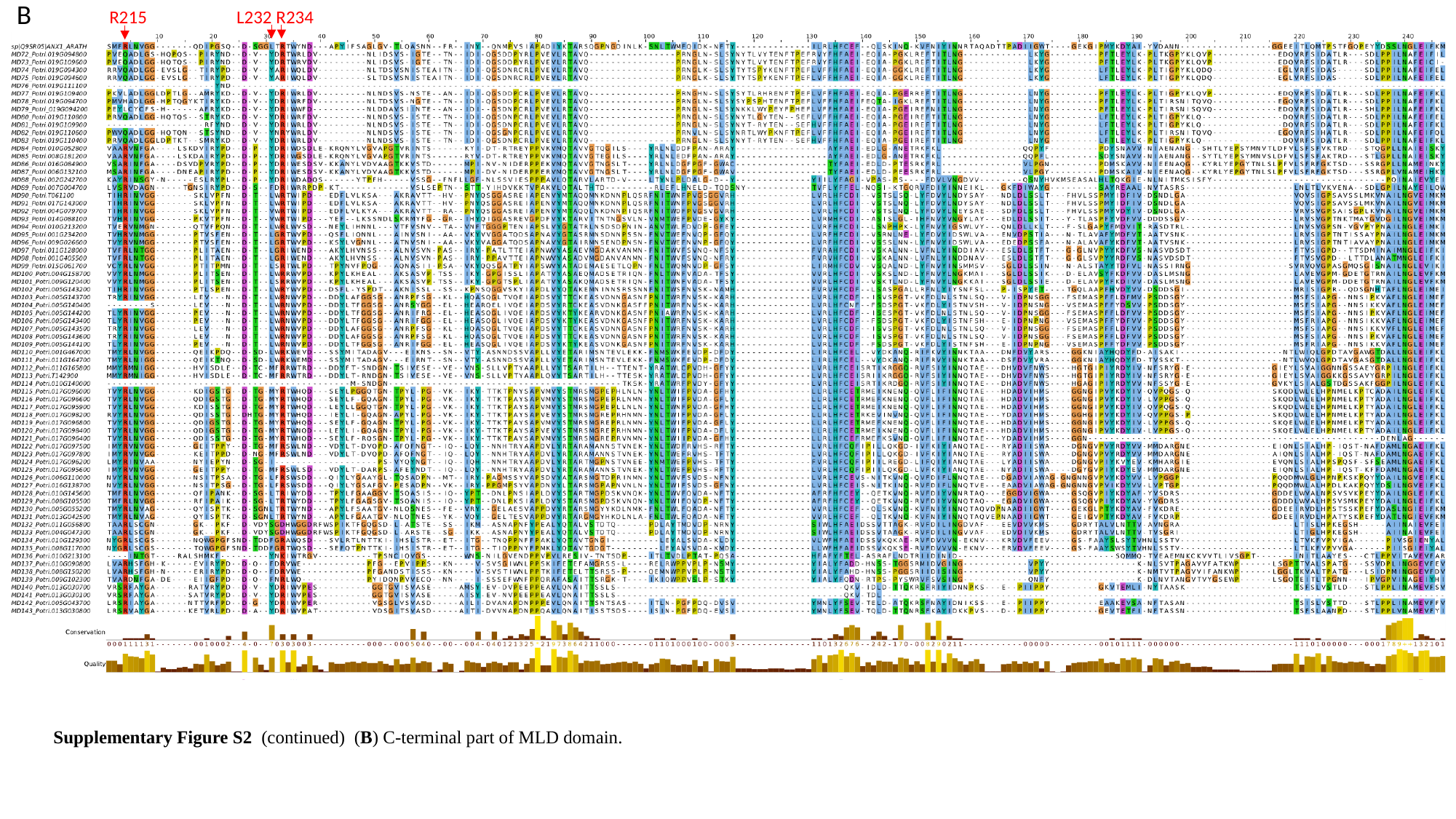

B
L232 R234
R215
Supplementary Figure S2 (continued) (B) C-terminal part of MLD domain.
