# Supplementary material for: Genome-Wide Identification of Populus Malectin/Malectin-Like Domain-Containing Proteins and Expression Analyses Reveal Novel Candidates for Signaling and Regulation of Wood Development
Source: Front Plant Sci. 2020 Dec 22;11:588846. doi: 10.3389/fpls.2020.588846 (PMC7783096; doi:10.3389/fpls.2020.588846)
Supplement: Supplementary file 8 [file Data_Sheet_1.PDF]

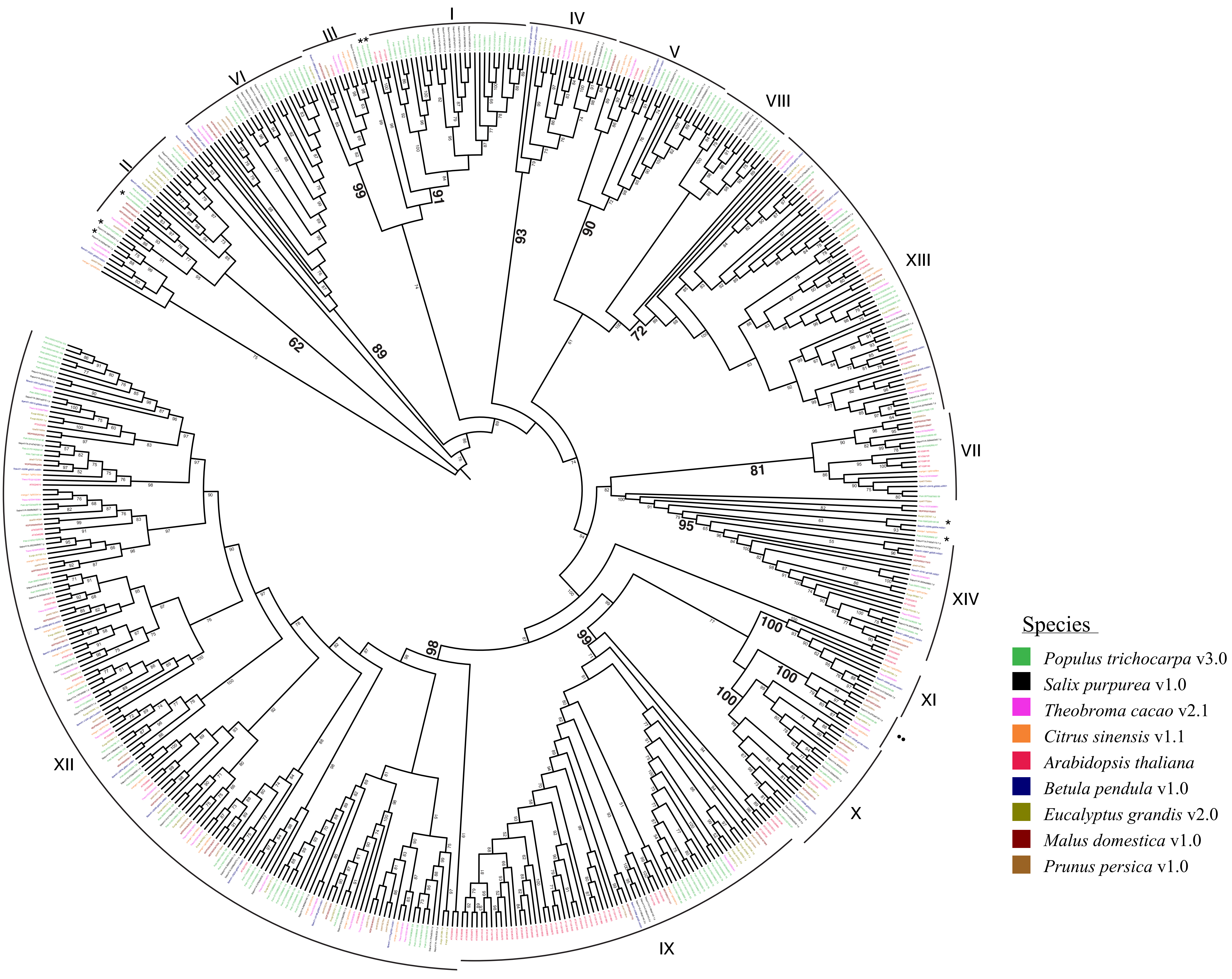

Supplementary Figure S3 Phylogenetic tree of MD proteins of different tree species and *Arabidopsis thaliana*. Bootstrap values (%) were shown only when larger than 50%. *P. trichocarpa* proteins are labeled with MD number after the Potri ID. *Pt*MDs with different clade association than that shown in Figure 1 and 2 were marked by asterisks. Double black dots denote *Pt*MD89 and its orthologs in other species.
